# Biology of *Rhynchaenus maculosus* provides insights and implications for integrated management of this emerging pest

**DOI:** 10.1038/s41598-022-18954-7

**Published:** 2022-08-27

**Authors:** Ruisheng Yang, Pengcheng Qiu, Yujian Gu, Mingyang Ni, Zhenhai Xue, Jianhua Han, Yiren Jiang, Ying Jin, Yong Wang, Xinfeng Zhou, Wei Liu, Jihui Zhang, Li Qin

**Affiliations:** 1 College of Bioscience and Biotechnology, Shenyang Agricultural University, Shenyang, 110866 Liaoning China; 2 Jilin Provincial Sericulture Institution, Agriculture Committee of Jilin Province, Jilin, 132012 Jilin China; 3 Hulunbuir Institute of Agriculture and Husbandry, Hulunbuir Agriculture Husbandry Bureau, Hulunbuir, 021000 Inner Mongolia China

**Keywords:** Zoology, Entomology

## Abstract

*Rhynchaenus maculosus* is an emerging insect pest with an increasingly serious tendency. Lack of biology information results in the bottleneck of integrated management of this pest. To facilitate an available design of integrated pest management strategy, biology of *R. maculosus*, including voltinism, life cycle, distribution, and damage has been investigated. Results reveal that *R. maculosus* is oligophagous and distributes in Heilongjiang, Jilin, and Liaoning provinces, China. This pest produces one generation per year (univoltinism) and overwinters as adults in leaf litter. From mid-April to late-April, active overwintering adults emerge from overwintering sites. The next generation of adult *R. maculosus* appears from mid-May to early June until mid-August to early September when the beetles move into the overwintering places. The entire time span of adult occurrence ranges from 315.6 ± 3.6 to 336.4 ± 3.2 days (Mean ± SD). Larvae undergo 3 instars with a total duration of 20 to 23 days. *R. maculosus* larvae feed on *Q. wutaishanica* and *Q. mongolica* without host-specific preference between the two host species, but do not harm *Q. acutissim*. Three species of larval parasites were collected and identified as Braconidae sp., Eulophidae sp*.*, and Ceraphronidae sp*.* Biological information of *R. maculosus* provides essential insights for design and implementation of integrated management of this pest.

## Introduction

The oak flea weevil *Rhynchaenus maculosus* (Coleoptera, Curculionidae, Rhynchaeninae), is an emerging pest of oak in China^1,2^. It was first discovered and described in 1991 as a new insect species collected in 1987 from Greater Xing'an Mountains, Heilongjiang province^3^. Since then, there had been no further reports of *R. maculosus* globally until spring of 2012 when the sporadic blister-like blotches were found on the leaves of *Q. wutaishanica* in the Oak Germplasm Resource Bank located in Shenyang Agricultural University, Shenyang, Liaoning, China (123° 34′ E/41° 50′ N). Initially, these symptoms were minor and misdiagnosed as oak freezing damage or disease. Therefore, the negative impact on forestry, horticulture, and oak sericulture did not attract enough attention of the researchers. In the spring of 2015, the weevil adults were found and collected in the blister-like blotches, and identified as the species *R. maculosus* in 2016. Currently, studies of *R. maculosus* mainly focused on morphology and species identification^1–3^. Biological information of this pest is extremely scarce with basic research progressing slowly. Application research such as comprehensive prevention and control is impossible to be addressed, resulting in a gradual increase in population and more severe damage. Eventually, the first outbreak occurred in Jilin, China in 2020, exhibiting a more serious tendency.

Biology is key for design of integrated pest management (IPM) strategies, especially for novel pests. To date, the *R. maculosus* outbreak only occurs locally in China. Therefore, biological research is of great practical significance for preventing as well as predicting the spread of this pest. In this study, biology of *R. maculosus*, including geographic distribution, life history, and hazards, were investigated and identified, aiming to provide detailed insights for its population spreading, prediction, and the formulation of IPM.

## Methods

### Investigation time and locations

**Figure 1:**
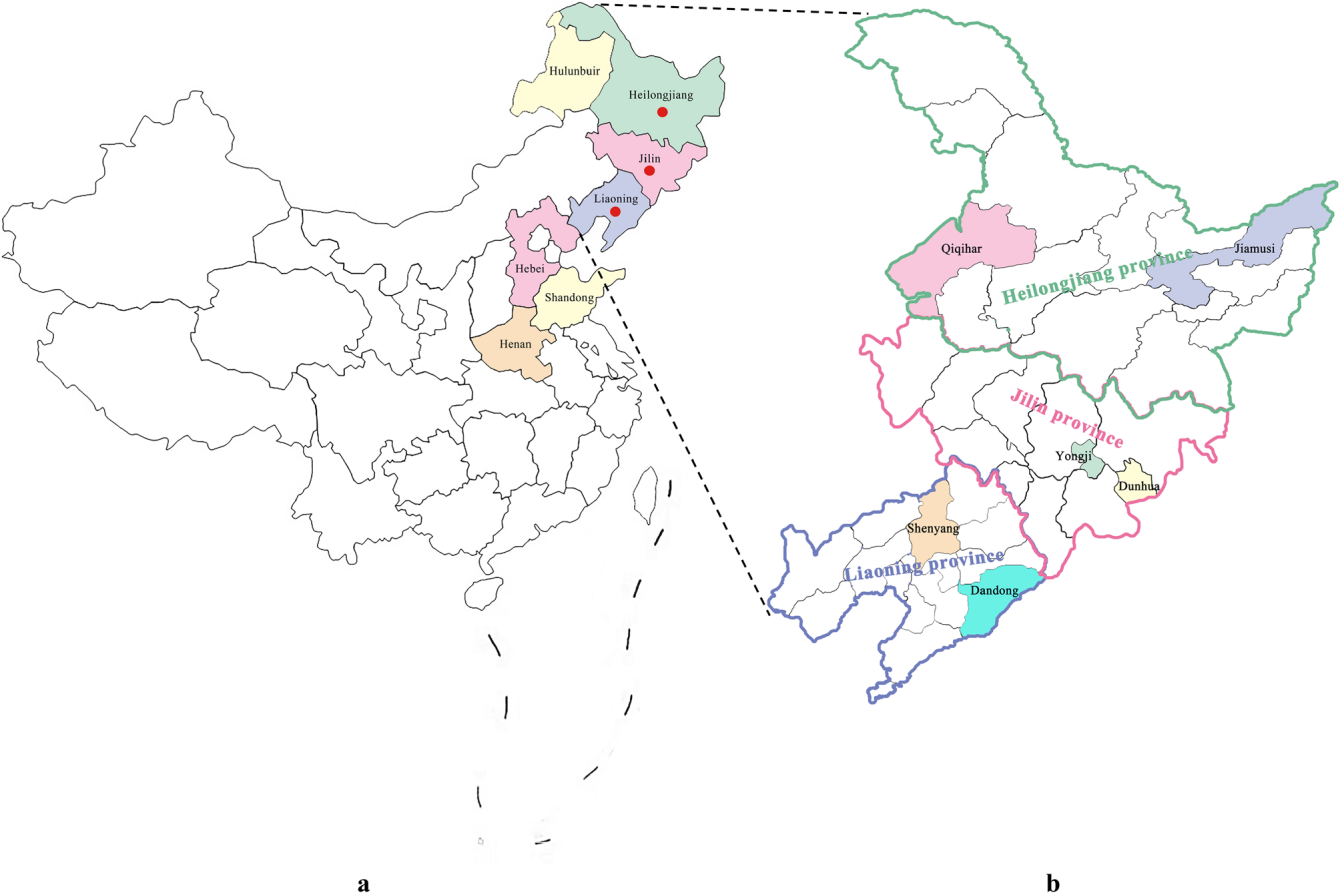
Maps of locations used in this study. (**a**) Map of locations used for distribution investigation. Red circles indicate the distribution areas of *Rhynchaenus maculosus*; (**b**) Map of locations used for biology investigations. The maps were created in QGIS version 3.26.0 available at https://qgis.org/en/site/.

From 2017 to 2021, surveys of the pest distribution were conducted in the regions of Inner Mongolia, Heilongjiang, Liaoning, Jilin, Hebei, Henan, and Shandong provinces (Fig. 1a), which are the main distribution areas of oak in China. Studies of life cycle and biology of *R. maculosus* were performed in Qiqihar (123°10′E/47°19′) and Jiamusi (130°22′E/46°47′N) (Heilongjiang province), Yongji (126°28′E /43°39′N) and Dunhua (128°16′E/43°23′N) (Jilin province), Shenyang (123°34′ E /41°50′ N) and Dandong (124°2′E/40°25′N) (Liaoning province) (Fig. 1b). The survey of dynamics of overwintering adult from 2017 to 2021 was carried out in Jiamusi, Heilongjiang province, Yongji, Jilin province, and Shenyang, Liaoning province, respectively. Investigations on larval damage on oak leaves were performed in the Oak Germplasm Resource Bank (123° 34′ E/41° 50′ N) located in Shenyang, Liaoning. Additionally, larva individuals used for instar determination were also collected from the Oak Germplasm Resource Bank.

### Distribution

Three oak forests over 100 km apart in each province were selected to collect the insect pests. The collection of adult samples was performed using sticky traps around the trunk combined with hand-collection from mid-April to early May each year^2^. The insect samples were taken into laboratory for species identification carried out by both morphology and molecular method.

### Life cycle and biology

To investigate the life cycle and biological characteristics of the larvae, pupae, and adults of *R. maculosus*, an oak forest with an area of 10 hectares was selected as a survey plot in each region with *R. maculosus* infestation. For this, twenty oak trees damaged by *R. maculosus* as well as 500 blister-like feeding spots were marked in the east, south, west, north, and the centre of each plot respectively*.* The biological characteristics of eggs were mainly investigated indoors combined with the field observations. After overwintering, active adult individuals were brought to the laboratory and fed with *Quercus wutaishanica* leaves for copulating and egg-laying in plastic boxes (10 cm*10 cm*5 cm) where the egg-laying was observed and egg duration measured. The dimensions of ten individuals in each stage were measured and recorded under a stereoscopic microscope. For egg load and fecundity surveys, active overwintering adults were field-collected by hand at Oak Germplasm Resource Bank located in Shenyang (Liaoning province). The collection was performed since mid-April until early May in 2020 and 2021. Fifty individuals of virgin female adults stored in 75% ethanol were dissected for the egg load assessment under a stereomicroscope. At the same time, another fifty female adults after copulation were collected and reared singly in a plastic tube (5 cm long × 1 cm diameter) for fecundity survey. A tender oak leaf was provided and changed regularly for the egg-laying in each tube until the female adult died. The plastic tube was placed under a stereoscope microscope to observe and count the laid eggs.

### Occurrence dynamics of active overwintering adults (Spring adults)

A square oak forest with an area of 5 hectares was selected from each distribution province. A five-point sampling method was adopted for each plot^4^. Taking the intersection of the diagonal lines of the square as the center sampling point, the respective vertices of the square were used as four sampling points. At each sampling point, two oak trees with a distance of three meters were selected. For each oak tree, a rectangular sticky trap (20 cm × 25 cm) around the trunk at 10 cm high above the ground was used for capturing the active overwintering adults^2^. The number of active adults stuck by the trap was surveyed at the same time each day from mid-April to mid-May in Liaoning and Jilin, from late-April to late-May in Heilongjiang. The active adults stuck on the traps were removed with tweezers to avoid affecting the effectiveness of insect-trapping.

### Timespan of adult occurrence

At the final stage of pupa, the observation on eclosion was carried out every day in the oak forest where the biology investigation was performed at the Oak Germplasm Resource Bank located in Shenyang. From 2017 to 2021, the timespan record of adult occurrence started from the day when the first new generation adult (summer adult) was found to the day when the active overwintering adult (spring adult) disappeared.

### Hunger tolerance of adult

*Rhynchaenus maculosus* adults were reared in an artificial climate box (LRH-250-GS) at 20–25 °C, with a humidity of 70%, and 21-h light a day. In the oak forest different from others used in this study, 200 spring adults and 200 summer adults were collected for starving experiment. Every five individuals as a group were reared in a plastic box (10 cm*10 cm*5 cm) with water in it provided by a saturated wet cotton wool. No food was provided for the adults to completely starve the pests^6^. The daily observation was carried out to record the dead insects for analyzing the lifespan of spring and summer adults respectively.

### Rate of leaf damage caused by larvae and host preference

From late April to early May, three oak forests, *Q. wutaishanica, Q. mongolica,* and *Q. acutissima* forests were used to assess the host preference in the Oak Germplasm Resource Bank located in Shenyang Agricultural University. At each oak forest, three square plots at a distance of 200 m from each other were selected as replications. According to the five-point sampling method^4^, a total of ten oak trees were selected from each plot. In each canopy, 10 branches were selected from the North, South, East, West and the center to record the number of damaged leaves and the total leaves for assessment of the damage rate of leaves in each plot. A significance analysis was performed using the statistics software SPSS to analyze the host preference among the different oak species ^5^.

### Determination of larval instar

From mid-April to mid-May in 2021, the larvae individuals (n = 401) were collected in batches and brought to the laboratory. The head capsule of each larva was removed and head capsule width (HCW) was measured and recorded under a stereo microscope.

Based on the frequency distribution of HCW, multi-peak normal fitting was performed using the Origin 2021 software (https://www.originlab.com/). Each single peak represented a larval instar and the intersection of two adjacent normal curves was considered as the dividing point of two instars. The number of instars acquired from analyzing the HCW frequency distribution was verified by the coefficient of variation, Brooks’ index^7,8^, and Crosby index^8,9^. The coefficient of variation less than 15% and Crosby index less than 0.1 were regarded as the criteria.

$$Brooks^{\prime}index = \frac{{X_{n} }}{{X_{n - 1} }}\quad Crosby^{\prime}s\; index = \frac{{b_{n} - b_{n - 1} }}{{b_{n - 1} }}$$

where *x*_*n*_ refers to the mean value of measurement indices for n-instar larvae, *x*_*n*-1_ refers to the mean value of measurement indices for n-1 instar larvae, *b*_*n*_ refers to the Brooks’ index of n-instar larvae, and *b*_*n*-1_ refers to the Brooks’ index of n−1 instar larvae.

### Natural enemies

In mid-May every year, oak leaves with mature larvae in blister-like blotches (pupa cell) were collected for parasite-culture (temperature = 25 °C, humidity = 70%, natural light) in a plastic insect-rearing box (20 cm × 20 cm × 15.5 cm). Three hundred larval individuals (blister-like blotches) from each distribution area were reared and examined respectively to investigate the parasitisation rates of *R. maculosus* larvae by parasite collecting, sorting, and species identifying.

## Results

### Life cycle and life history

**Figure 2:**
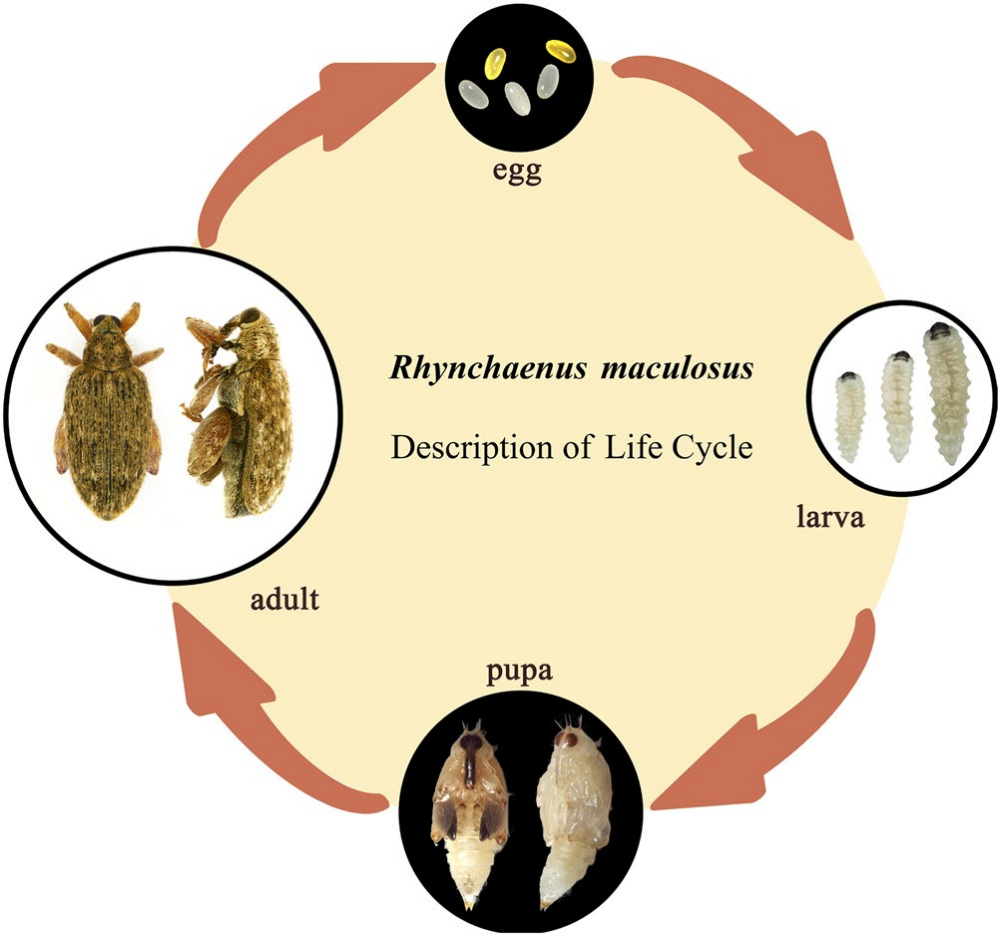
Life cycle of *Rhynchaenus maculosus*. Egg: early-stage eggs have a milky-white color whereas yellow indicates the late-stage; Larva: the three larval individuals from left to right indicate the 3 instars of the whole larval stage; Pupa: ventral view to the left and lateral view to the right; Adult: dorsal view to the left and the lateral view to the right.

*R. maculosus* undergoes a complete metamorphosis and develops through four distinctive developmental stages, namely egg, larva, pupa, and adult stages (Fig. 2).

**Figure 3:**
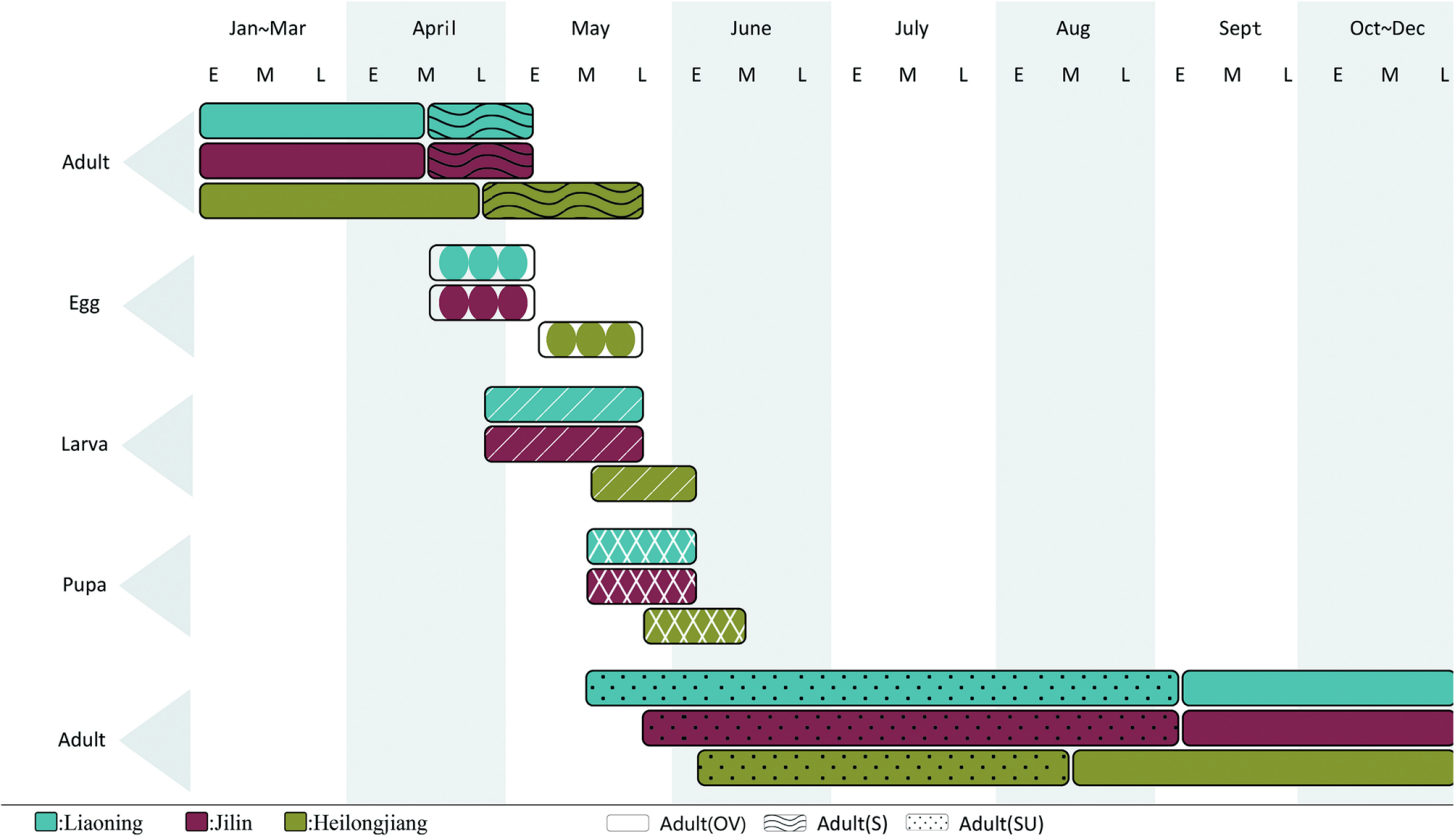
Life history of *Rhynchaenus maculosus* in Liaoning, Jilin and Heilongjiang provinces. Capital letters OV denote overwintering adult, S denotes spring adult and SU denote summer adult.

**Figure 4:**
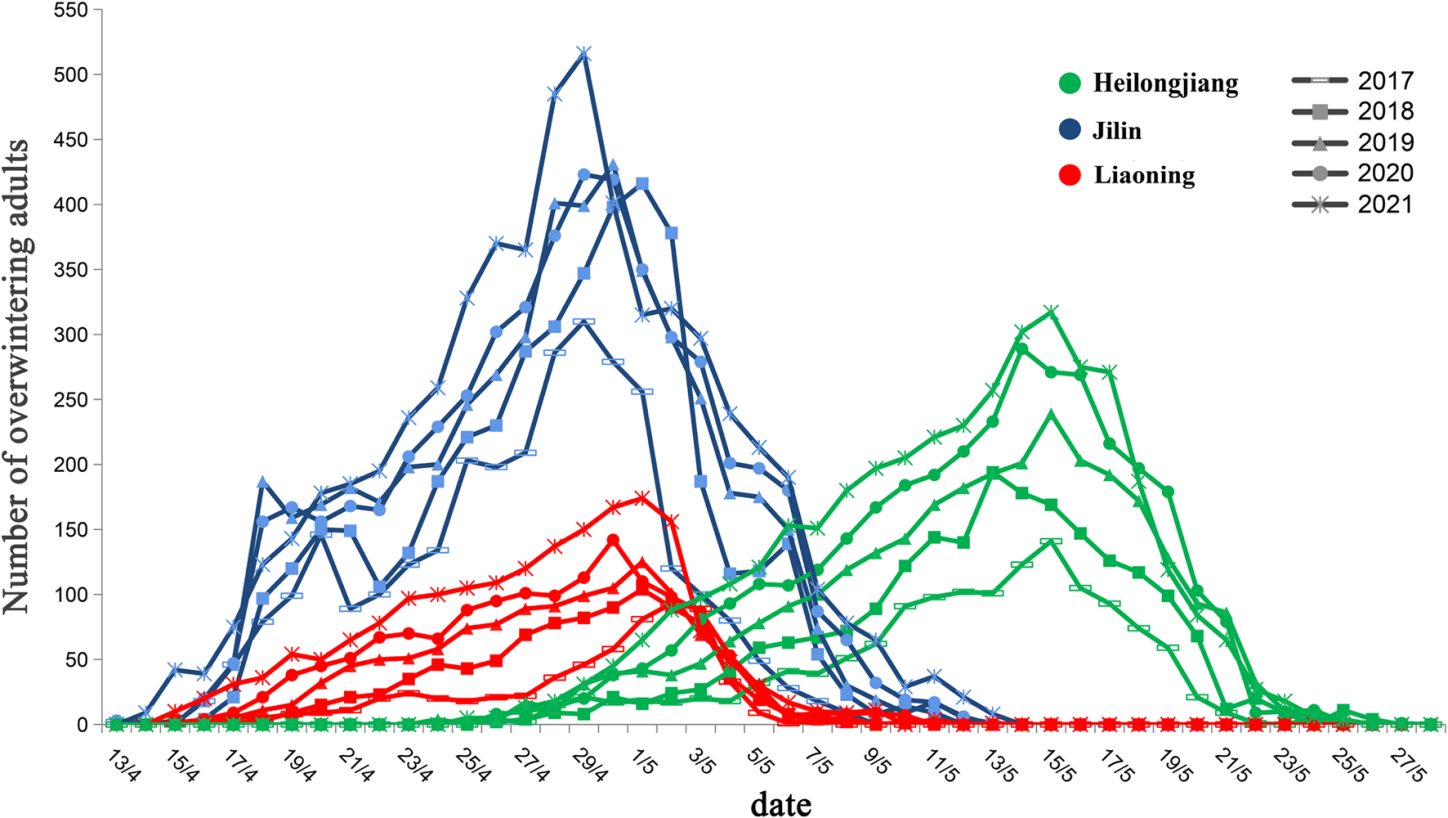
Dynamics of active overwintering adult (spring adult) occurrence of *Rhynchaenus maculosus* in different locations.

This pest has one generation per year with univoltine and overwinters as adult stage in the leaf litter or topsoil under the tree canopy, or in the rolled and withered leaves on the oak in Liaoning, Jilin, and Heilongjiang provinces, respectively. Active overwintering adults (spring adults) emerge from hibernation sites at different times depending on the distribution locations (Fig. 3). In Liaoning and Jilin province, the spring adults terminate the hibernation and emerge from their overwintering sites in mid-April to feed on the tender buds and leaves of host oak. The peak period of spring adult activity in Liaoning and Jilin is almost synchronized, both ranging from 28th April to 2nd May, and from 13th May to 15th May in Heilongjiang. After the emergence peak, there is a sharp decrease in the number of spring adults until the adults gradually disappear in early May in Liaoning and Jilin, and late May in Heilongjiang with a whole duration of 25–28 days in all three locations (Fig. 4).

The female spring adults deposit their eggs on the second day after mating. Eggs take about 4–5 days to hatch into larvae. The newly hatched larvae burrow into the oak leaves at the end of April in Liaoning and Jilin, and in mid-May in Heilongjiang, and cause damage by leaf mining in the mesophyll during the whole larval stage. The larvae develop into mature stage after 20–23 days and pupate gradually from mid-May to late May in Liaoning and Jilin, and in late May in Heilongjiang. Adults of the next generation (summer adults) emerge in mid-May in Liaoning and late May in Jilin province and continue to feed on the oak leaves until early September when they start seeking overwintering sites both in Liaoning and Jilin. In Heilongjiang province, the spring adults occur at the end of April for feeding, copulating, and egg-laying, with subsequently hatching and feeding on leaf mesophyll in larval stage. The larvae mature and pupate from late May to mid-June. The summer adults appear in early June and continue to feed on the oak leaves until they terminate feeding and start overwintering in mid-August (Fig. 3).

### Biology

**Figure 5:**
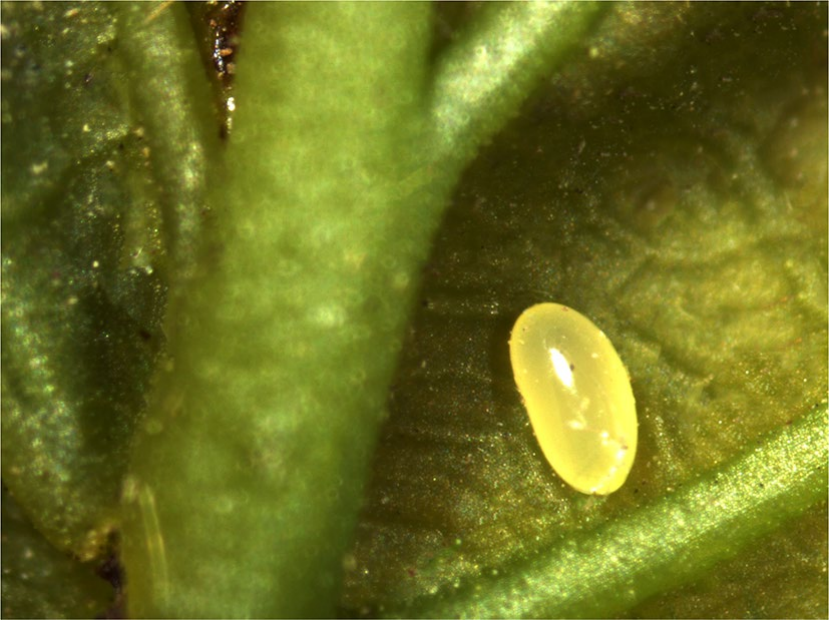
Single egg of *Rhynchaenus maculosus* deposited near the midvein on the lower surface of the oak leaf.

The egg has a smooth surface and is elliptically shaped with a long diameter of 0.5 to 0.6 mm and a short diameter of 0.3 to 0.4 mm. The newly oviposited egg is milky white and gradually turn yellow before hatching. Eggs are deposited in mid-April both in Shenyang and Dandong, Liaoning province, Yongji and Dunhua, Jilin province, and early May in Qiqihar and Jiamusi, Heilongjiang province, with duration of 4-5 days. The majority of eggs oviposited singly are scattered near the veins on the lower surface of the leaves with the minority on the upper surface (Fig. 5). The egg load of an adult female ranged from 7 to 13 and the number of eggs laid by an adult female was 5–10 in its lifetime.

**Figure 6:**
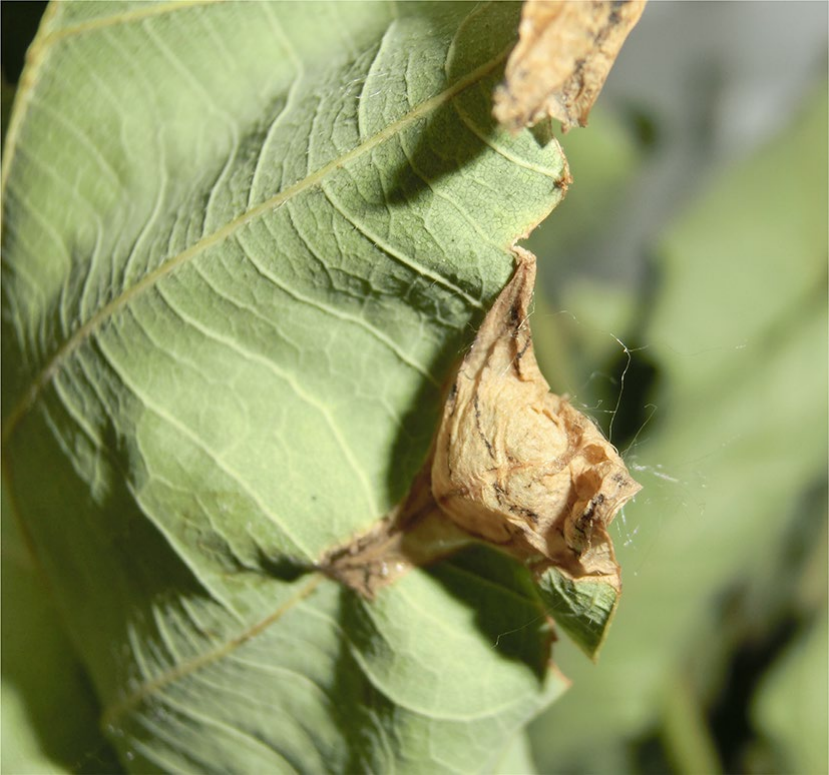
Blister-like blotch (pupa chamber) on the edge of an oak leaf.

The larva is milky white, flat, and footless with obvious segmentation. The mature larvae are 5.5–6.2 mm long. The newly hatched larvae emerge in late April in Shenyang, Dandong, Yongji and Dunhua, and early May in Qiqihar and Jiamusi. After hatching, the tiny larvae directly burrow into the leaf epidermis and feed on the mesophyll inside the oak leaves, forming irregular blister-like and yellow–brown blotches. With the increase of instar, the larvae feed gradually towards the leafedges, expanding the blotches, with the largest diameter of the leaf blotch reaching 3–4 cm. The larvae gradually mature in mid-May in Shenyang and Dandong, Liaoning province, Yongji and Dunhua, Jilin province, and in early June in Qiqihar and Jiamusi, Heilongjiang province (Fig. 3). During the mature larva stage, a hollow blister-like blotch (pupa chamber) is formed on the edge of oak leaf (Fig. 6). The larval stage takes 20–23 days with three instars.

The pupa is exarate in type and spindle-shaped with sparse spines. Mature larvae pupate in the pupa chamber at the leafedge with pupa duration of 6–8 days.

The adult *R. maculosus* is spindle-shaped and covered with dense gray hair. The mouthparts are snout-shaped and opisthognathous in type. The adults exhibit a pair of well-developed hind legs which allow jumping movements.

**Figure 7:**
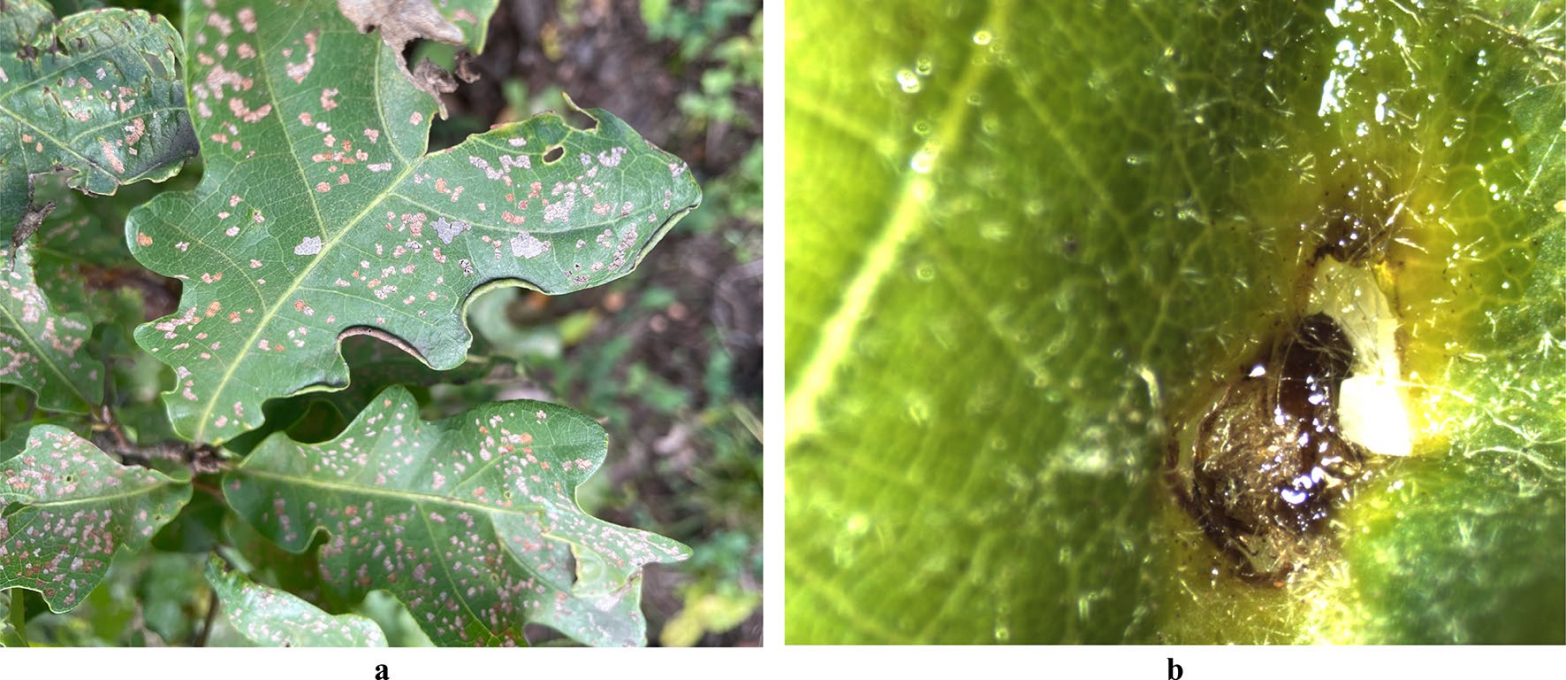
Damage symptoms caused by adults of *Rhynchaenus maculosus.* (**a**) Damage caused by summer adults; (**b**) Damage caused by spring adults.

The time of summer adult occurrence varies with the different distribution areas. Summer adults emerge in mid-May, late-May, and early June in Liaoning, Jilin and Heilongjiang provinces, respectively (Fig. 3). Adult eclosion occurs within the pupa chambers (blister-like blotches) from 23:00 pm to 1:00 am, with an emergence rate of 98% in the wild. After emergence, the summer adult does not immediately exit the pupa chamber but stays within for additional 1–2 days before biting through the pupa chamber. After emerging, the summer adults continue to damage the oak leaves by feeding on the upper epidermis and leaf tissues of oak leaves, forming dense and irregularly-shaped blotches (Fig. 7a). The adult *R. maculosus* is prominently characterized by well-developed meta-legs with strong ability to jump. In case of interference, the adults exhibit a strong ability to jump about 50 cm at a time and poor capacity of long-distance flying. The summer adults stop feeding and overwinter in the leaf litter or topsoil under the canopy, or in the rolled and withered leaves in the canopy in early September both in Liaoning and Jilin, and in mid-August in Heilongjiang.

After overwintering, the active spring adults (spring audlts) emerge from the hibernation sites for feeding, mating on the winter buds or tender leaves. The copulation lasts for about two hours. Interestingly, the damage symptom caused by spring adults is different from that by summer adults. Spring adults only causes slight damage to oak trees by piercing the tender buds (or leaves) and sucking the sap with the snout-shaped mouthparts, forming irregular tiny holes on tender leaves (Fig. 7b). The occurrence of adult *R. maculosus* has a entire time span of 315*.*6 ± 3.6–336.4 ± 3.2 days (Mean ± SD), including a feeding period of 110.4 ± 2.0–115.0 ± 2.5 days (Mean ± SD) and an overwintering period of 205.2 ± 2.3–221.0 ± 1.6 days (Mean ± SD).

The adult *R. maculosus* exhibits a strong ability of hunger tolerance. At temperatures between 20 and 25 °C, spring adults can survive for 3 to 6 days without water and food, while the summer adults slightly longer, 4 to 8 days.

### Determination of larva instar

**Figure 8:**
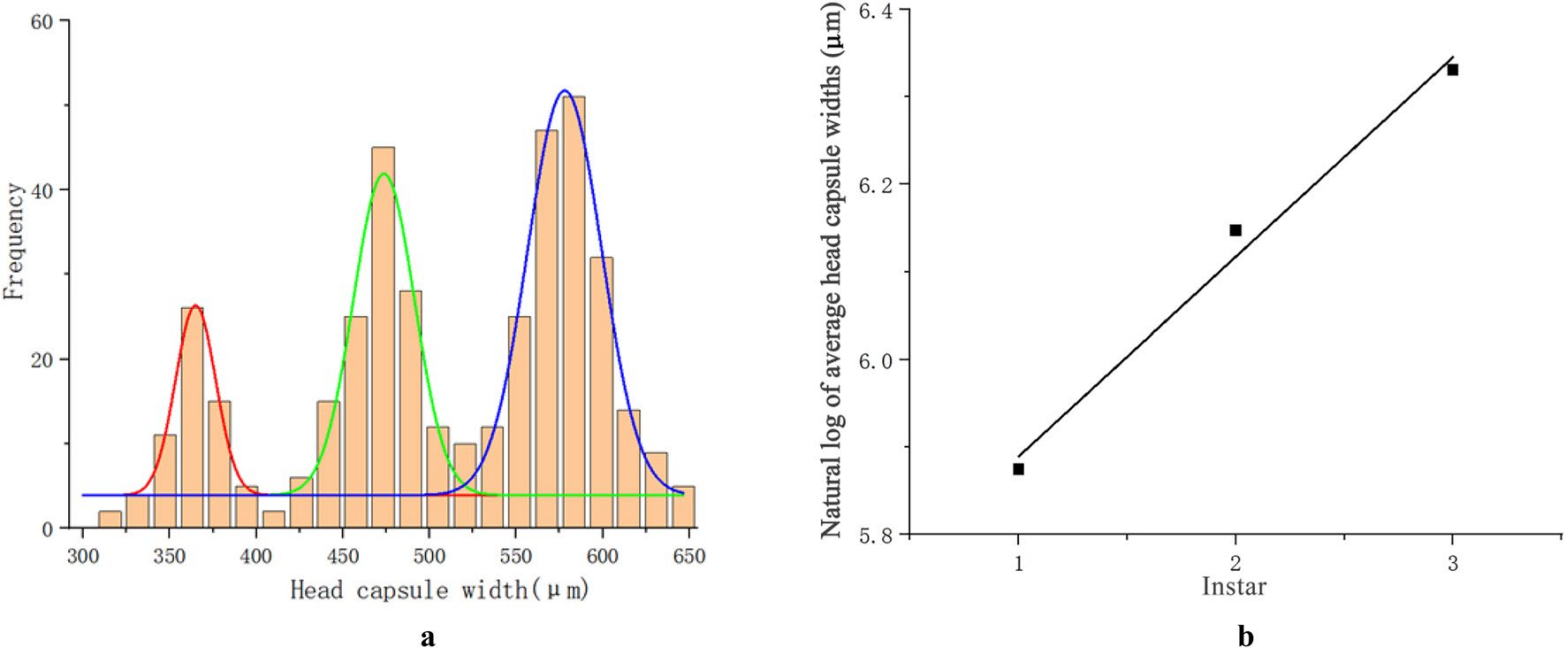
Determination of larval instars of *Rhynchaenus maculosus*. (**a**) Frequency distribution histogram of head capsule width in different larval instars; (**b**) Linear regression between the natural logarithms of average head capsule widths and larval instars.

Analysis from multi-peak normal fitting distribution showed three obvious peaks of the head capsule width (HCW) indicating three instars in the whole larval stage (Fig. 8a). Each intersection point of the two normal fitting curves reveals a two instar cut-off point.

The HCWs of *R. maculosus* larvae range from 300 to 650 μm (n = 401). Crosby’s indices for the average values of HCWs measured in different instars were less than 0.1, and the coefficients of variation for the morphology data measured from each instar are less than 15% (Table 1), resulting in the determination of three instars during the larval stage. There is a highly significant relationship in the linear regression between the natural logarithms of mean HCW values and instars (n = 3, R^2^ = 0.994, F = 170.82, *p* = 0.05) (Fig. 8b). The results strongly supported the determination of three instars in the whole larval stage of this pest.

**Table 1:** Average values, coefficients of variation, Brooks indices, and Crosby’s indices of the head capsule widths (HCWs) from three larval instars of *Rhynchaenus maculosus*.

Instars	Mean ± SE/μm	Coefficients of variation/ *%*	Brooks indices	Crosby’s indices
1	365.0 ± 1.8 (n = 65)	5.30		
2	473.7 ± 1.3 (n = 145)	5.26	1.2978	
3	578.0 ± 1.1 (n = 191)	4.38	1.2202	−0.0598

### Distribution and host plant

**Figure 9:**
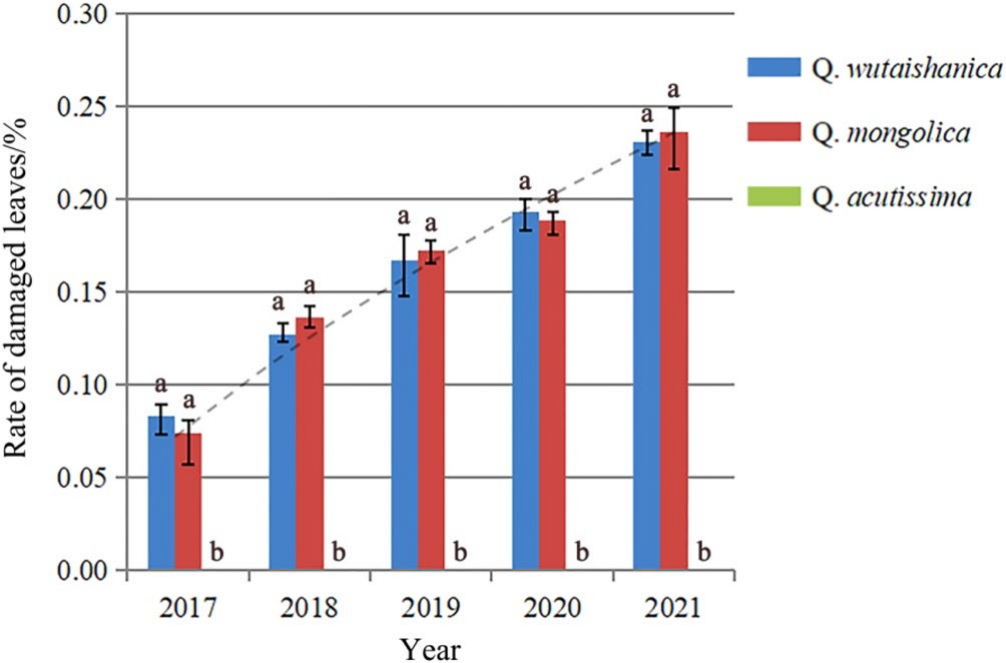
Host plant preference analysis and damage trend of *Rhynchaenus maculosus* larva from 2017 to 2021. Significant difference of host preference is indicated by the letters a and b; The dotted line indicates the damage trend of pest larvae.

Investigations revealed that *R. maculosus* is distributed in Heilongjiang, Jilin, and Liaoning provinces, China (Fig. 1). This pest is oligophagous in feeding and only feeds on oak (genus *Quercus)* in its larval and adult stage, such as *Quercus wutaishanica* and *Q. mongolica*. Analysis based on the leaf damage rate revealed no significant difference between *Q. wutaishanica* and *Q. mongolica*, suggesting no host preference between the two host plant species during the larval period. There was a remarkable increase of the damage rate to the leaves of the two *Quercus* species from 2017 to 2021, while no damage on *Q. acutissima* has been found yet (Fig. 9).

### Natural enemies

**Figure 10:**
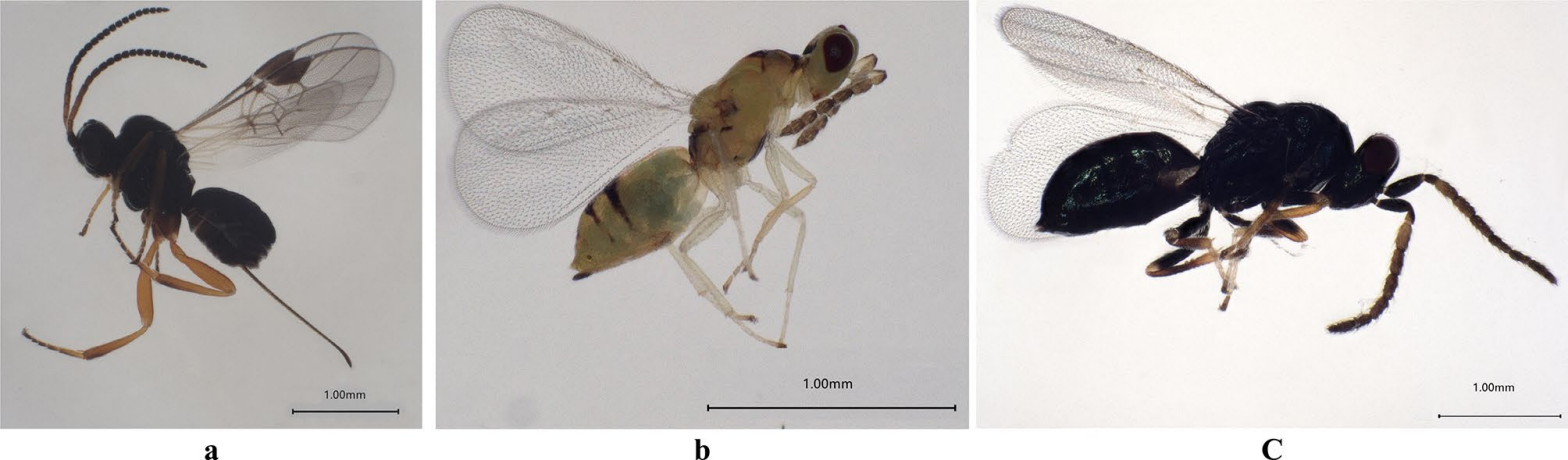
Three species of parasitoids cultivated from mature larvae of *Rhynchaenus maculosus*. (**a**) Braconidae sp. collected in Liaoning; (**b**) Eulophidae sp. collected in Liaoning; (**c**) Ceraphronidae sp. collected in Jilin and Heilongjiang.

A total of three parasitoid species (Hymentoptera) were observed and collected from mature larvae or pupae in the pupa chambers including Braconidae sp. and Eulophidae sp. in Liaoning, and Ceraphronidae sp. both in Jilin and Heilongjiang (Fig. 10). Most maggots of these parasitoids emerge in late May with the *R. maculosus* larvae developing into their mature stage. Data from 2017 to 2021 revealed average parasite rates for Braconidae sp. of 1.3%, Eulophidae sp. of 2.0%, and Ceraphronidae sp. of 3.7% in Jilin and 4.7% in Heilongjiang, respectively.

## Discussion

Biology like life cycle and life history is the basic attribute of individual development for an insect pest, which plays a crucial role in pest monitoring and provides significant insights and implications for the effective pest control^10,11^. In the present study, the results suggested that *R. maculosus* undergoes four stages, egg, larva, pupa, and adult in its lifetime. The biological profiles of this pest provided critical clues for the design and implementation of integrated pest management strategy.

For the egg stage, there are several factors that make *R. maculosus* infestation less effective or difficult to control with the conventional chemical methods. For example, in similarity to other flea weevil species^12,13^, in the present study the majority of eggs were laid near the veinson the lower surface of the leaves that can’t be treated easily with traditional pesticide spraying mode. Another factor making the pest infestation less effective to control in the egg stage is its single oviposition on leaf. Therefore, egg is not considered as an appropriate stage and shouldn’t be taken into consideration in the integrated pest control of *R. maculosus*.

Larva, the second stage of life cycle, is one of the most damaging stages and therefore crucial for *R. maculosus* control. The present study revealed that most *R. maculosus* larvae in the 1st and 2nd instar stages occur from late April to early May in Liaoning and Jilin provinces and from mid-May to late May in Heilongjiang province. Therefore, this is the most critical period for this pest control, because the larvae in the 1st and 2nd instars are most sensitive to insecticides and can be controlled much easier than at later instars. In addition to the control period, the insecticide mode of action is another issue to consider in *R. maculosus* larva control. Insect pests in ‘protected’ microhabitats, such as feeding under the bark or wood, below ground, or even in leaf tissue, will be protected to a greater or lesser extent from insecticide or climatic extremes^14,15^, resulting in an increasing pest population. *R. maculosus* larvae live their entire lives protected within the oak leaf tissue by feeding on the mesophyll inside the oak leaves, which make it difficult, even impossible to effectively treat larvae with the contact insecticide. Therefore, a systemic insecticide would perform a prominent efficacy against the leaf-mining larvae due to its systemic and foliar penetration properties^16,17^. For the larvae of *R. maculosus* would be directly exposed to systemic insecticide throughout its whole larval stage inside the oak leaves.

In the implementation of integrated pest management program, adult control is an indispensable factor for regulating pest populations. Adults are the most active among the four stages with the complexity of their behavioral activities and physiological functions, which makes it difficult to design and implement an adult control program, and meantime provide a possibility for the development of adult control tactics^18^. Our study showed that the occurrence of the overwintering adults (spring adults) in different distribution areas had an obvious peak period. The peak period in Liaoning and Jilin provinces was from 28 April to 2 May and between 13 and 15 May in Heilongjiang Province, which revealed the exact period for spring adult control. In addition, the investigation showed that the adults overwintered in the leaf litter under the host canopy, and most of them emerged from the overwintering sites, climbed up the trunk to feed on the buds or tender leaves when the host tree germinates. This information provides at least two options for controlling spring adults. First, during the emergence from the overwintering sites, the leaf litter under canopies can be sprayed by conventional spraying method using contact insecticides, which would effectively kill the most of the active spring adults. Second, sticky trap around the base of trunk can be used to prevent the spring adults from climbing up the trunk. An effective control of spring adults would greatly reduce the possibilities of egg laying, which contributes to population reduction of the next generation. The occurrence period of summer adults varied with the distribution areas. In Liaoning and Jilin, the summer adults emerged in mid-May and late May respectively and overwintered gradually in early September. In Heilongjiang, the summer adults emerged in early June until mid-August they gradually hide themselves in the overwintering places. This information provides crucial clues for the control period of summer adults.

Distribution of an insect pest is one of the population properties, which is of great significance either in theoretical ecology as well as in pest monitoring and control^19^, especially for a newly-emerging pest. Our studies showed that *R. maculosus*, a newly-emerging insect pest in recent years^2^, is distributed locally in Northeast China, including the Liaoning, Jilin, and Heilongjiang provinces. Some leaf-mining pests in the weevil family have become invasive pests, causing economic and ecological impacts to the newly introduced areas^20^. In case of *R. maculosus,* early monitoring and intervention are essential in preventing its potential establishment or spread to other areas in China or even globally. Despite the limitation of its self-mediated spread capabilities in our study, the possibility for *R. maculosus* of a vector-borne spreading and becoming an invasive pest in other regions of China or the world remains to be seen.

Besides insecticide-dependant control, pest control by natural enemies plays a significant role in the regulation of pest populations and is considered as an ecologically and economically promising solution^21^ for IPM. In this study, three species of parasitoids emerged from *R. maculosus* larvae, with the natural parasitisation rates of 1.3% (Braconidae sp*.*) in Liaoning, 2.0% (Eulophidae sp.) in Liaoning, and of 3.7% (Ceraphronidae sp.) in Jilin, 4.7% (Ceraphronidae sp.) in Heilongjiang. Although the parasitism rate is not high and far from being an effective pest control for *R. maculosus*, at least the natural enemy provides the possibility of an economically and ecologically promising solution in *R. maculosus* management. At present, we have only investigated the parasitic natural enemies in the larval stage. As for the following issues, whether the larvae are parasitized by other parasite insects or pathogens, whether there are natural enemies in the adult, pupa and even egg stages, and how the different natural enemies manipulate the pest population effectively, there are still much for us to do for the successful IPM.

In agreement with other flea weevils^13,22^, *R. maculosus* undergoes one generation per year (univoltinism) and overwinters as adults in all distribution areas. While the issues like life history, occurrence dynamics, and natural enemy information vary with the different geographic distributions and underlie the establishment of IPM for *R. maculosus*. An effective design and implementation of IPM strategies would, therefore, be adjusted to these different biological parameters of this pest.

The knowledge of biology provides guidance for the integrated management of *R. maculosus*. However, there is still much to do in its further study such as physiology (e.g. resistance to host secondary metabolites), chemical ecology (e.g. attractants), molecular biology (e.g. function genes), and IPM. In addition, *R. maculosus* only infests the *Quercus* plant in the field, which implies that the host volatiles are a potential attractants. Meanwhile, identification and application of sex pheromone of female adult could contribute to the development of an alternative and eco-friendly method for controlling this pest.

## Data Availability

All data used and analyzed during the current study are available from the corresponding author on request.
